# Job Insecurity and Job Performance: A Serial Mediated Relationship and the Buffering Effect of Organizational Justice

**DOI:** 10.3389/fpsyg.2021.694057

**Published:** 2021-09-09

**Authors:** Marco De Angelis, Greta Mazzetti, Dina Guglielmi

**Affiliations:** ^1^Department of Psychology “Renzo Canestrari”, University of Bologna, Bologna, Italy; ^2^Department of Education Studies “Maria Giovanni Bertin”, University of Bologna, Bologna, Italy

**Keywords:** job insecurity, organizational justice, work-family conflict, burnout, performance, moderated mediation

## Abstract

The study aimed to extend the current knowledge of the relationship between job insecurity and performance. In line with traditional stress theories, work-family and burnout were hypothesized as serial mediators of the negative link between job insecurity and job performance. Also, the current study hypothesized that the association between job insecurity and the mediators [i.e., Work-family conflict (WFC) and burnout] could be buffered by perceived organizational justice among employees. Therefore, we empirically tested a moderated serial mediation model. Participants were 370 employees of an Italian multiservice social cooperative. Data were collected using a self-report questionnaire in the aftermath of the COVID-19 pandemic outbreak. The obtained results indicated that WFC and burnout mediated the association between job insecurity and job performance. Furthermore, perceived organizational justice buffered the relationship between job insecurity and WFC. Concerning job burnout, the association with job insecurity was moderated only among employees perceiving medium and high levels of organizational justice. The moderated serial mediation index provided support to the role of organizational justice in decreasing the association between job insecurity and job performance. This study delves deeper into the variables explaining the relationship between job insecurity and job performance by testing a serial process mechanism that involved WFC and burnout. Additionally, the obtained results provide suggestions to organizations and managers regarding the protective role of organizational justice to sustain employees’ mental health and performance. Practical implications at the organizational and managerial level are provided, along with a focus on the actual impact of the pandemic.

## Introduction

Recent years and mainly the 21st century have profoundly affected the labor market worldwide. Economic, technological, societal, and political upheavals have increasingly undermined the concept of secure employment (Yeves et al., 2019). For instance, an increasing number of employees work outside the traditional workplace, just as work schedules are gradually flexible, making the boundaries between work and personal life increasingly blurred and complex to manage (Gerstel and Clawson, 2018).

In other words, a gradual change in basic assumptions toward work-life flexibility has led to significant changes in working conditions that, in turn, have fueled greater job insecurity (Benach et al., 2014). The recent COVID-19 pandemic has further exacerbated this condition, bringing anxieties and concerns about one’s professional and financial future to the surface (Wilson et al., 2020). This insecurity enhanced the level of distress and concern among workers about their job and financial futures (Menéndez-Espina et al., 2019). Also, the damage caused by the pandemic in several employment sectors and the rising levels of unemployment (Blustein et al., 2020; McKibbin and Fernando, 2020) put additional pressure on employers and organizations in terms of being both competitive and responsible for preserving the health and performance of their employees (Wilson et al., 2020; Rasdi et al., 2021). Overall, such changing working conditions require a better understanding of how employees respond to such changes and the consequences for employees’ psychological and physical health and job performance.

Job insecurity is defined as the perceived fear of losing the current job for unexpected and uncontrollable events that can interrupt the continuity of one’s work experience (Sverke et al., 2002; De Witte, 2005; Shoss, 2017). During the last few years, job insecurity has received significant interest from academic research due to changes in the labor market and organizational settings. Unpredictable economic environments and increased market competitiveness have led to company downsizing and reorganization, thus increasing the perceived insecurity of employees, who are worried about losing their jobs and concerned about finding new job opportunities (Sverke et al., 2002).

Furthermore, job insecurity has received growing attention due to its impact on workers’ mental health, wellbeing, and organizational performance (Sverke et al., 2006; Shoss, 2017). For example, job insecurity is negatively associated with job satisfaction, organizational commitment, and wellbeing (Sverke et al., 2002; Berntson et al., 2010; Green, 2011), which indicates the stressor role of job insecurity. Furthermore, the harmful outcomes of job insecurity include burnout symptoms, a conflict between one’s job role and personal life, and a significant reduction in life satisfaction (Richter et al., 2010).

In terms of work-related stress, the JD-R model (Bakker and Demerouti, 2014, 2017; Schaufeli and Taris, 2014) allows framing job insecurity as a stressful job demand that can deteriorate psychological health and individual energies if not balanced with adequate work-related resources (Mauno et al., 2007). Job insecurity may cause negative consequences in employees’ wellbeing, attitudes toward their job, and behaviors at work. However, research focusing on behavioral outcomes, especially on employee performance at work, is still limited.

Meta-analytic evidence reported the negative impact of job insecurity on task performance (Sverke et al., 2002; Cheng and Chan, 2008; Gilboa et al., 2008). However, while many previous studies have concluded that job insecurity has a negative effect on task performance explicitly, some mixed empirical findings can be found (e.g., Lee et al., 2018; Debus et al., 2019; Pilipiec, 2020; Shin and Hur, 2020). The inconsistency of these findings suggests the need for a closer look at this phenomenon. Recently, Stankevičiūtė et al. (2021) found that job insecurity harmed both task performance and organizational citizenship behavior. Also, Piccoli et al. (2021) investigated the effect of job insecurity on job performance, hypothesizing a two-dimensional stressor framework where both hindrance and challenge effects were considered. The result of the two studies conducted supported the negative relationship between job insecurity and job performance.

Therefore, building on previous research findings, we developed the first hypothesis as follows:

*Hypothesis 1 (H1):* Job insecurity would be negatively related to job performance.

In the literature, the focus has often been on highlighting the harmful effects of job insecurity on both personal health and job attitudes and outcomes. However, the link between job insecurity and job performance is still unclear (Stankevičiūtė et al., 2021). Therefore, focusing on exploring the potential mechanisms underlying the link between job insecurity, health, and job outcomes through intermediate drivers seems to be an area of research that needs to be addressed (De Witte et al., 2016).

This focus becomes even more crucial when considering the need for managers to gain a more comprehensive understanding of the job-insecurity-performance relationship to develop organization-wide strategies that can prevent stress reactions and support individual and organizational effectiveness (Piccoli et al., 2021). Therefore, the purpose of this study was twofold: first, to provide a conceptual framework that identifies possible individual psychological mechanisms underlying the effect of job insecurity on employee performance and second, to hypothesize a possible organizational response that protects against the adverse effects of job insecurity that could recommend concrete responses for managers and practitioners.

### The Mediating Role of WFC and Burnout

Gaining a deeper understanding of workers’ experience of job insecurity and its consequences on psychological health and job performance has become crucial. So far, the literature has been focused on exploring the relationship between job insecurity on mental health outcomes (e.g., László et al., 2010; Griep et al., 2021) from one side, work attitudes, such as job satisfaction (Di Stefano et al., 2020), counterproductive work behavior (Van den Broeck et al., 2014), and job performance (e.g., Stynen et al., 2015) on the other side. However, there is a growing focus on exploring the underlying mechanisms of how job insecurity develops into subsequent health and behavioral effects through intermediate drivers (De Witte et al., 2016). In other words, a specific concern is to understand the conditions under which job insecurity leads to impaired performance (Di Stefano et al., 2020).

Based on the resource-based model of stress (Lazarus and Folkman, 1984), several studies have considered job insecurity a stressor that results in poor mental health outcomes. A recent meta-analysis suggested the deteriorating negative role of job insecurity on individuals’ physical and mental health (Jiang and Lavaysse, 2018). In line with these results, studies have tried to link the impact of job insecurity on job outcomes, such as performance through levels of individual wellbeing. Darvishmotevali and Ali (2020) investigated how job insecurity affects employees’ subjective wellbeing and job performance in the hospitality industry. They found a mediating role of subjective wellbeing, affirming that job insecurity negatively impacts employees’ job performance *via* decreasing their subjective wellbeing.

Furthermore, these results show that employees with a high level of psychological capital can cope with job insecurity. Parent-Lamarche et al. (2021) explored the mediating role of employees’ wellbeing in the association between organizational conditions and job performance. Job insecurity was indirectly associated with lower professional efficacy and job performance levels through a negative association with employees’ wellbeing. The results based on the stress-related mechanism highlighted the need for targeted changes in working conditions.

According to the JD-R model (Schaufeli and Taris, 2014), persistent exposure to excessive job demands (i.e., job insecurity) may trigger symptoms of emotional exhaustion that, in the long run, may result in detrimental individual and job-related outcomes (e.g., an impaired job performance). Consistent with the health-impairment process, the enduring experience of job insecurity could engender a condition of chronic emotional exhaustion (e.g., burnout) and eventually translate into harmful outcomes for individuals and their work environment, thus deteriorating job performance.

In exploring the impact of job insecurity at the individual level, previous research has focused on understanding the potential consequences in the private life sphere. Indeed, the anxiety and fears of losing a job and the related economic impact have negatively affected the work and family domains. Work-family conflict (WFC), also called work-family interference, has been defined as a type of inter-role conflict that occurs when job demands and family needs are perceived as incompatible (Byron, 2005). Empirical findings suggest that the negative relationship between job insecurity and subjective wellbeing was partially explained by a greater occurrence of WFC (Hu et al., 2018).

Previous studies provide evidence for the spillover effect of job insecurity on WFC. While investigating the long-term impact of perceived job insecurity, Rocha et al. (2006) had highlighted adverse effects on workers’ mental health and their families’ wellbeing, who were at risk of experiencing various stress-related problems due to the individuals’ pressure faced from lack of control over their job future. Also, Richter et al. (2010) highlighted how workers who experience job insecurity also reported WFC, particularly among men. Interestingly, a recent systematic review on the consequences of job insecurity on family-related outcomes (Mauno et al., 2017) outlined the different theoretical mechanisms underlying this relationship. The relationship between job insecurity and WFC would thus be traced to direct or indirect spillover effects. Experiences and events occurring in different domains (e.g., increased workload due to fear of losing one’s job) can mutually influence each other (e.g., difficulty managing family commitments, raising one’s children, and marital tensions). In this sense, the job preservation motivation strategies and proactive coping strategies (Shoss, 2017; Giunchi et al., 2019) could further explain the mechanisms underlying people’s active efforts in work. In other words, when faced with the possibility of losing their job, people decide to devote more attention, effort, and energy to preserving their job or developing alternative strategies, such as finding a new job. Accordingly, the risk is that people may have to take time and resources away from their family duties, thus negatively affecting the work-family balance. In other complex cases, the spillover turns into a crossover effect, in which the discomfort experienced by the worker is transferred to his/her family members in a kind of contagion (e.g., empathy). Recent studies have highlighted the potential negative impact of pandemic-induced work changes in the family environment (Fisher et al., 2020). School closures, working from home, and the need to attend to family duties are some examples that may suggest how the pandemic has severely affected the family domain, particularly in terms of time conflict where work and home hours proceed simultaneously (Rudolph et al., 2020). Against this background and in a climate of profound uncertainty about the future, work-family balance can therefore become critical to workers’ wellbeing and performance.

In conclusion, while the evidence of job insecurity and WFC on individual’s mental health is strong (De Witte et al., 2016; Jiang and Lavaysse, 2018; Griep et al., 2021), the implications of the relationship between job insecurity, WFC, and employee’s mental health on work outcomes (i.e., job performance) still need to be clarified.

Based on the existing related theories and research, the present study assumes that the subjective experience of job insecurity and perceived actual working situation implies various employees’ reactions to a similar uncertain employment condition (De Witte et al., 2015). As recently found by Piccoli et al. (2021), people can cope with uncertainty passively, with adverse effects on their health and performance, or proactively, thus finding new energy sources to improve their work performance. The results, however, showed that job insecurity does not lead to reactive coping strategies and that, therefore, the direct and indirect impacts on job performance are likely to be negative.

The evidence reported so far confirms that experiencing WFC can result from high job insecurity (Richter et al., 2010; Mauno et al., 2017). Employees who perceive a threat to their employment will react accordingly by devising strategies to safeguard their job position or seeking alternatives. In other words, if not managed appropriately, these coping strategies risk deteriorating personal resources and thus the work-family balance. Given the negative impact of both job insecurity and WFC on individuals’ mental health (Mutambudzi et al., 2017; Griep et al., 2021), this study aims to advance the literature on job insecurity by hypothesizing a potential underlying mechanism in which intermediate drivers (WFC and mental health) could explain the negative impact of job insecurity on job performance. Therefore, our model hypothesized how this health-impairment process triggers coping strategies that ultimately turn out to be maladaptive (Piccoli et al., 2021), such as deteriorating workers’ mental health and job performance.

Hence, the second and third hypotheses of the current study were developed as follows:

*Hypothesis 2 (H2)*: WFC and job burnout mediate the relationship between job insecurity and job performance.

*Hypothesis 3 (H3)*: WFC predicts burnout in the serial mediation between job insecurity and job performance.

### The Buffering Role of Organizational Justice

Job insecurity has also been studied from a theoretical social exchange perspective (Shoss, 2017). Employees perceive a threat to their job position due to the perceived imbalance in their exchange relationship with the organization. In other words, job insecurity might result from the mismatch between the individual’s investment in the organizational environment (e.g., performance) and the perceived fair treatment employees receive from the organization. Several studies have explored the relationship between job insecurity and psychological contract violations through the lens of organizational justice theory (Sora et al., 2010, 2021; Piccoli and De Witte, 2015). One of the core mechanisms underlying the negative impact of job insecurity on employees’ wellbeing entails the breach of the psychological contract (defined as the perceived mutual obligations between two parties, the employee and the employer) and the perceived lack of fairness in organizational processes and decisions, with the latter having a greater weight in explaining the negative impact on employees’ feelings of emotional exhaustion (Piccoli and De Witte, 2015).

Accordingly, Sora et al. (2021) found that job insecurity was indirectly related to self-rated performance through the three types of organizational justice, namely, distributive (the perceived equity in terms of quality of the outcomes provided by the organization), procedural (the perceived fairness on how decisions are taken, and results are assigned), and interactional justice (refers to the perceived fairness in the social exchange with the organization and managers). This study also highlighted how these relationships varied depending on the type of contract.

Informational justice is defined as the perceived adequacy of organizational and managers’ strategies employed in sharing information about implemented organizational decisions, processes, and outcomes. This component of organizational justice can balance the resources lost as a consequence of job insecurity. Schumacher et al. (2020) examined how job insecurity relates to job performance based on the Conservation of Resources theory. The authors assumed a reduced impact of job insecurity on employees’ performance when exposed to greater levels of informational justice. Employees reported lower levels of contextual performance and productivity during the weeks they experienced higher levels of job insecurity than usual, except where the organization had adequately informed employees of any significant upcoming changes. In the latter case, contextual performance and productivity levels remained intact.

The impact of organizational justice on how employees balance their work commitments, family duties, and responsibilities has also been studied. Indeed, work-related experiences and perceptions can negatively affect employees’ life outside the workplace. Not surprisingly, several aspects of the work environment result in significant interference with the family (Grandey et al., 2007). In this sense, employees perceiving an unfair working environment where personal duties and constraints (e.g., family responsibilities) are not adequately considered could suffer from an unsatisfactory work-family balance. In line with previous research (Kyei-Poku, 2014; Sánchez et al., 2020), WFCs are more likely to occur when the organization and the way managers treat their employees are not perceived as fair. In other words, it is mainly from an interpersonal and informational perspective that the concept of organizational justice assumes its relevance with work-family balance. On the contrary, in a working environment where the organizational management pays attention to the way employees are treated and justifies their decisions by sharing information openly and transparently, employees could better manage their work demands and thus have more resources available to manage private or family commitments.

Based on the job insecurity-related literature, the concept of organizational justice has often been assumed to play a mediating role, thus explaining the underlying reason for adverse work-related behaviors (Shoss, 2017; Sora et al., 2021). However, several studies have found that organizational justice can also moderate the negative effects of job insecurity (Lee et al., 2018). For example, Silla et al., (2010) highlighted how an organization perceived as fair and equal moderates the adverse effects of job insecurity on aspects, such as commitment, satisfaction, or intention to stay. Following the theoretical perspective of social exchange, Di Stefano et al. (2020) showed how a supportive organizational context moderated the relationship between job insecurity, employee-leader relationships, and job-related outcomes, such as job satisfaction and turnover intention.

Drawing on the concept of balancing job demands and resources, Bakker and Demerouti (2014) describe the direct and moderating processes that job resources can have on the health-impairment process. Several studies have showed that both personal and job resources can mitigate the impact of job demands. Also, considering the pandemic’s impact on both individuals and organizational performance, some job-related and organizational factors could play a crucial role in exacerbating or moderating people’s mental health (Giorgi et al., 2020).

**Figure 1:**
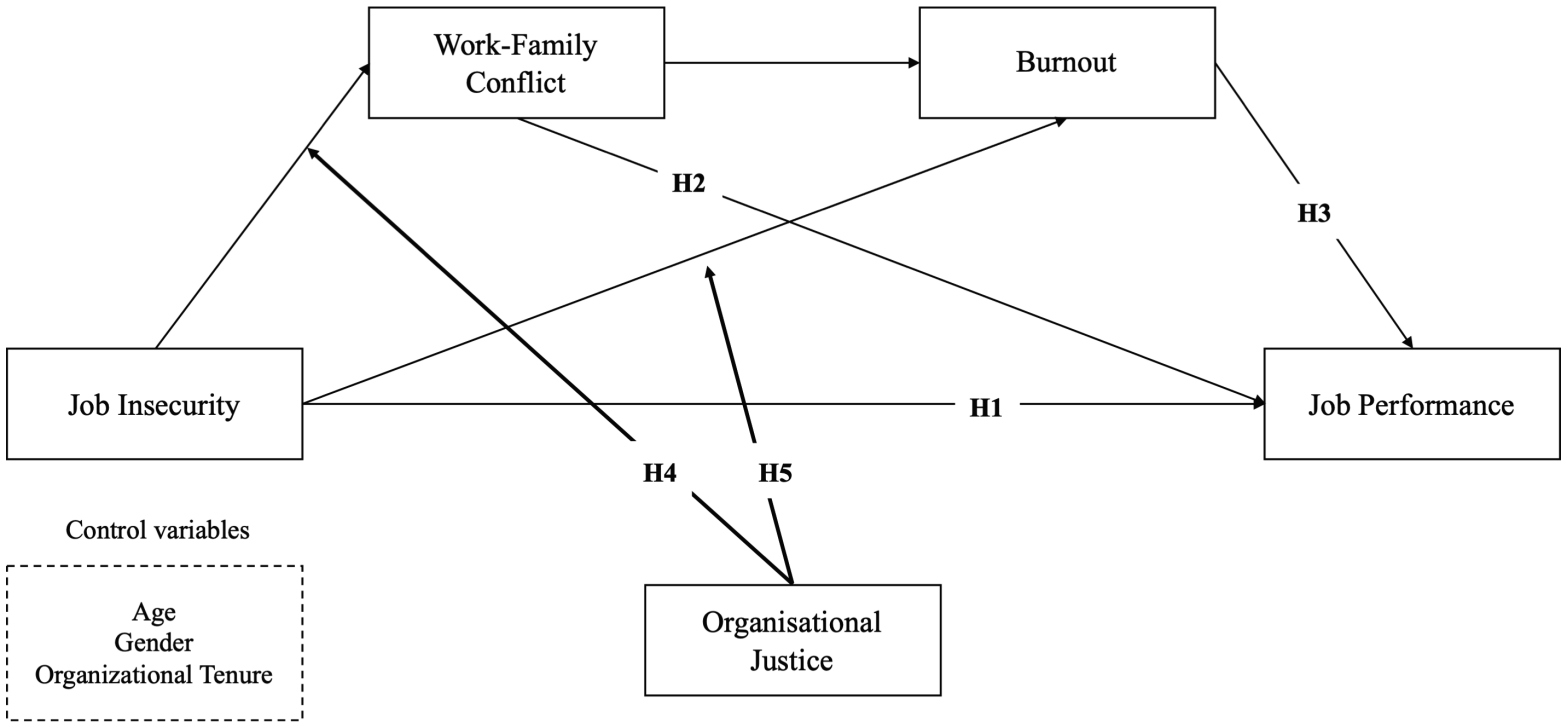
Hypothesized moderated serial mediation model.

In the present study, we sought to further advance the literature on job insecurity by investigating how the role of a perceived fair organization can moderate the stress–strain process triggered by job insecurity, thereby preserving employees’ work-family relationships, mental health, and, ultimately, job performance. In this sense, drawing on the impact that organizational justice may have on the emotional and mental health of individuals and their work-family balance, the current study hypothesized a moderating role of organizational justice on the health-impairment process, which, in turn, might hamper the adverse effects of job insecurity on job performance. In other words, perceived organizational justice might represent a contextual boundary condition (i.e., a moderator) of the indirect relationship between job insecurity and the two mediators with job performance. A moderated mediation model (Figure 1) was tested to examine whether the indirect effect of job insecurity on performance through WFC and burnout would be more considerable for employees who perceived a lower level of organizational fairness. Thus, we hypothesized that as:

*Hypothesis 4 (H4)*: Perceived organizational justice would moderate the effect of job insecurity on WFC. In other words, employees perceiving a high level of organizational justice are expected to experience a lower WFC when compared to those reporting a lower level of perceived organizational justice.

*Hypothesis 5 (H5)*: Perceived organizational justice would moderate the effect of job insecurity on burnout. To be specific, we hypothesize a more significant occurrence of burnout symptoms among employees perceiving low levels of organizational justice when compared to those characterized by a higher perception of organizational justice. Figure 1 displays the hypothesized model.

## Materials and Methods

### Procedure

Data were collected between December 2019 and January 2020 using an online questionnaire as part of a psychosocial risk assessment project among employees from an Italian multiservice cooperative. The cooperative is multi-sectoral, operating in the social and educational fields, managing cultural heritage, and communication and information. Participation in the questionnaire was voluntary.

This research study arose from the opportunity to collect data as part of a psychosocial risk assessment required by the company to comply with national legislation (d. lgs. no. 81/2008). In this sense, it was not necessary to request approval from the University Ethics Committee. However, the questionnaire’s first page summarized the study’s contents and goal and reported the informed consent form, emphasizing participant anonymity and information confidentiality. This document complied with personal data treatment guidelines defined by the Italian privacy law (Law Decree DL-196/2003) and the GDPR, Regulation 2016/679. Participants had to select the consent checkbox at the end of this page as a prerequisite to access the questionnaire.

The cover letter also declared that the employer would not be informed of participants’ decision not to complete the survey. Concerning the ethical standards for research, the study complies with the latest version of Helsinki’s Declaration (World Medical Association, 2013).

### Participants

A total of 482 employees have accessed the survey link. Only questionnaires with a minimum of 70% of answers were retained. Hence, the final sample consisted of 370 employees. Table 1 provides a general overview of sample characteristics in terms of frequencies and descriptive analyses.

**Table 1:** Sample characteristics, descriptive, and frequencies.

	Frequency	Valid percent
**Gender**		
Female	275	74.3
Male	95	25.7
**Job contract**		
Permanent (full-time)	96	26.0
Permanent (part-time)	190	51.5
Temporary (full-time)	18	4.9
Temporary (part-time)	51	13.8
Temporary (other)	14	3.8
**Education level**		
Primary or lower secondary school diploma	15	4.1
Post-secondary school diploma	76	20.5
University degree	222	60.0
Post-graduate degree (Master’s degree, Ph.D., etc.)	57	15.4
**Supervisory role**		
Yes	74	20.7
No	283	79.3

Among them, 74.1% (*N*=274) were female, and 25.7% (*N*=95) were male.

The age of the participants ranged from 19 to 72years old. The mean age of the working population was 38years old (*SD*=10.03), the mean women’s age was 37.58 (*SD*=9.62), and the mean for men was 40.17 (*SD*=10.90). The survey also explored the job tenure of employees. On average, employees have been working for the current organization for 6years (*SD*=5.80), with a mean job tenure substantially equal across genders (women’s job tenure *M*=6.04years; *SD*=5.87; men’s job tenure *M*=6.11; *SD*=5.57).

Concerning the job contract, most of the sample (*N*=190; 51.4%) had a permanent part-time contract, 25.9% (*N*=96) had a permanent full-time contract, 13.8% (*N*=51) had a fixed-term part-time, 4.9% (*N*=18) had a fixed-term full-time contract, and the remaining 3.8% (*N*=14) worked as seasonal or temporal employees. Following De Cuyper and De Witte (2005), in the analysis, we recoded the job contract into a binary variable (i.e., 0=permanent; 1=temporary). Furthermore, frequency revealed that a small number of participants (20%) held a supervisory role regarding work positions. The working population involved in the survey has a bachelor’s degree (60.0%) or a higher qualification, such as a master’s degree or a Ph.D (15.4%).

### Measures

#### Socio-Demographic Characteristics

Participants’ gender (categorical variable), age, and job tenure (both as continuous variables) were asked to participants at the beginning of the questionnaire. These variables were included in the analysis as cofounding variables as previous studies suggested the link of age (Yeves et al., 2019), gender (Menéndez-Espina et al., 2020), job tenure (Cheng and Chan, 2008), and job contract (De Cuyper and De Witte, 2005) with job insecurity.

#### Job Insecurity

We used a 5-item self-reported scale developed by Chirumbolo and Hellgren (2003) to measure job insecurity. The scale was aimed to assess participants’ perceived chance of losing their job soon. Examples of items were, “I fear I will lose my job” and “I am concerned about keeping my job.” Participants expressed their accordance with each item with 5-point Likert scale ranging from 1 (*totally disagree*) to 5 (*totally agree*). The scale showed robust internal reliability (Cronbach’s alpha=0.81). Participants with higher scores expressed more serious concern about the chance of keeping their current work.

#### Work-Family Conflict

To measure WFC, we administered a 3-item scale based on the Italian work-related stress questionnaire developed to assess psychosocial risk factors and validated by Guglielmi et al. (2011). The items asked participants to state how frequently anxiety, worries, efforts, and time spent on their work negatively affect their family duties and responsibilities. Responses were provided with 5-point Likert scale ranging from 1 (*never*) to 5 (*very often*). Examples of items were, “I am so tired and stressed when I leave work that it is difficult for me to fulfill my family duties.” The scale showed robust internal reliability (Cronbach’s alpha=0.89). Participants with higher scores expressed more concern about being able to meet the demands of their private life.

#### Job Burnout

To obtain a measure of burnout, we used the short version of the Burnout Assessment Tool developed by Schaufeli et al. (2020); Italian version: Consiglio et al., 2021). In the present study, participants replied to burnout’s four core subscale dimensions: exhaustion, mental distance, cognitive impairment, and emotional impairment. Examples of items were, “After a day at work, I find it hard to recover my energy” (exhaustion), “I struggle to find any enthusiasm for my work” (mental distance), “At work, I have trouble staying focused” (cognitive impairment), “At work I may overreact unintentionally” (emotional impairment). Each item was rated with 5-point Likert scale ranging from 1 (*never*) to 5 (*always*). We used the composite score and compared the overall mean (*M*=1.99; *SD*=0.55) with the statistical norms for both Flemish and Dutch employees (Schaufeli et al., 2020) revealed a general average level of burnout among the working population. The scale showed robust internal reliability (Cronbach’s alpha=0.85). Participants with higher scores reported a greater occurrence of symptoms that might suggest experiencing burnout at work.

#### Organizational Justice

To obtain a measure of the perceived organizational justice, we used the Italian Organizational Health Questionnaire developed by Avallone and Paplomatas (2005). Accordingly, participants reported how managers and the organization treat, evaluate, and incentivize employees answering four items. Examples of items were, “The criteria for evaluating people are fair and transparent” or “Managers treat employees fairly.” Participants were asked to rate how often the scenario described by each item occur with 5-point Likert scale ranging from 1 (*never*) to 5 (*always*). The scale showed acceptable internal reliability (Cronbach’s alpha = 0.78). Participants with higher scores perceived their organization as fairer and more transparent in managing and evaluating their employees.

#### Job Performance

The overall perceived job performance was rated using the single item proposed by Shimazu et al. (2010): “On a scale from 0 to 10, where 0 is the worst performance, and 10 is the top performance, how would you rate your overall job performance during the past 4weeks?” with 11-point Likert scale ranging from 0 (*worst possible job performance a person could have on this job*) to 10 (*top job performance*).

### Statistical Analysis

The hypothesized model was tested using the PROCESS macro v3.5 (Hayes, 2017) on SPSS (version 23). Several steps were involved in the data analysis. Firstly, we examined the variables under investigation regarding their normality, kurtosis indices, and skewness. Also, means, standard deviations (SDs), Cronbach’s alpha, and bivariate correlation coefficients between the key variables were calculated to examine the association between all study variables (Table 2). Cohen’s guidelines allowed us to establish the magnitude effects (Cohen, 1988) for “small” (0.10), “medium” (0.30), and “large” (0.50) correlation effects. Next, using the PROCESS macro, it was possible to test the hypothesized models, the serial mediation, and the further moderated serial mediation model. The advantage of using PROCESS is to analyze the index of moderated mediation with simple soles results (standard error, *t*-value, and value of *p*), thus deepening the understanding of the relationship between variables. The serial mediation model was quantified to determine whether WFC mediated the effects of job insecurity on job performance and whether WFC and burnout serially mediated the relationship between job insecurity and job performance.

**Table 2:** Means, SDs, Cronbach’s alpha, and correlations of the variables used in the study (*N*=370).

	*M*	*SD*	Range	1	2	3	4	5	6	7	8	9
Age	38.28	10.0	–	–								
Gender (0=female)	0.26	–	0–1	0.11^*^	–							
Job tenure	6.08	5.79		0.42^**^	0.01	–						
Job contract (0=permanent)	0.22	–	0–1	−0.41^**^	−0.02	−0.45^**^	–					
Job insecurity	2.36	1.07	1–5	−0.03	−0.12^*^	−0.16^**^	0.33^**^	(0.89)				
Work-family conflict	2.48	0.94	1–5	−0.01	−0.09	0.04	0.12^*^	0.14^**^	(0.81)			
Job burnout	1.96	0.60	1–5	0.04	0.04	0.06	−0.12^*^	0.20^**^	0.63^**^	(0.85)		
Job performance	8.41	1.22	1–10	0.05	−0.08	−0.08	0.07	−0.10^*^	−0.26^**^	−0.38^**^	–	
Organizational justice	2.52	0.80	1–5	−0.10^*^	−0.10	−0.16^**^	0.23^**^	−0.08	−0.29^**^	−0.40^**^	0.26^**^	(0.78)

* *p<* 0.05;

** *p<* 0.01; ^***^p< 0.001.

Moreover, the model tested whether organizational justice moderated the association between simple and serial mediation effects. The moderated serial mediation model is based on three linear regression analyses (Hayes, 2015). In the first regression analysis, the first mediator (WFC) is predicted by the independent variable (job insecurity), the moderator (organizational justice), and the interaction between the independent and moderating variable (job insecurity × organizational justice). In the second regression analysis, the second mediator (burnout) is predicted by the independent variable (job insecurity), the moderator (organizational justice), their interaction (job insecurity × organizational justice), and the first mediator (WFC). Finally, in the third regression analysis, the dependent variable (job performance) is predicted by the independent variable, the moderator, their interaction, the first mediator, and the second mediator (Figure 1).

All variables in the model were centered before the analyses to compute the interaction terms. The interaction between the independent and moderator variables was examined with simple slope analyses (Aiken and West, 1991). In particular, the conditional effects were examined at low (mean - 1 SD), medium (mean), and high (mean + 1 SD) values of organizational justice. Indirect effects and the moderated mediation effect were assessed with 95% bias-corrected confidence intervals (CIs) based on 5,000 bootstrap samples (Hayes, 2015, 2017). This type of analysis allowed us to estimate the lower and upper CIs within which the indirect effect can be considered statistically significant (i.e., CIs different from zero).

## Results

Table 2 displays the means, standard deviations, internal consistencies (Cronbach’s alpha), and correlations between the study variables. All variables showed satisfactory reliability, with Cronbach’s alpha coefficients of 0.70 or higher.

Job insecurity was negatively correlated with gender (1=male), job tenure, overall job performance, and organizational justice, whereas it correlated positively with WFC and burnout. As expected, organizational justice revealed a significant negative correlation with all the study variables except for job insecurity.

The hypothesized model was tested using Model 6 described by Hayes (2017), in which an independent variable (i.e., job insecurity) is directly associated with the dependent variable (i.e., job performance) and indirectly associated through a serial mediation relationship (i.e., WFC and burnout, respectively). In this sense, the model explores both the direct effect of job insecurity on job performance and the indirect effect in a serial sequence. Table 3 displays the standardized regression coefficients, standard errors (SE), and model summary information for the hypothesized serial mediation model. In the model, age, gender, job tenure, and job contract were included as controlling variables.

**Table 3:** Regression coefficients, SE, and model summary information for the serial mediation model.

	WFC (M1)			Burnout (M2)			Job performance		
	*b*	SE	*p*	*b*	SE	*P*	*b*	SE	*p*
(Intercept)	2.46	0.24	< 0.001	0.90^***^	0.12	< 0.001	9.73^***^	0.36	<0.001
Job insecurity	0.24^***^	0.05	< 0.001	0.16^***^	0.03	< 0.001	−0.08	0.06	0.15
WFC	–	–	–	0.61^***^	0.03	< 0.001	−0.02	0.08	0.70
Burnout	–	–	–				−0.33^***^	0.15	<0.001
Age	−0.10^*^	0.01	0.01	−0.02	0.01	0.70	0.12^*^	0.01	0.04
Gender (0=female)	−0.06	0.11	0.26	0.10^*^	0.05	0.02	−0.08	0.14	0.14
Job tenure	0.04	0.01	0.50	0.04	0.01	0.40	−0.09^*^	0.01	0.11
Job contract (0=permanent)	−0.17^*^	0.15	0.01	−0.06	0.07	0.22	0.06	0.19	0.31
	*R*^2^ =0.07 *F*_(5,343)_ =4.942 *p* <0.001			*R*^2^ =0.43 *F*_(6,342)_ =42.825 *p* <0.001			*R*^2^ =0.16 *F*_(7,341)_ =9.357 *p* <0.001		

* *p<* 0.05; ^**^p< 0.01;

*** *p<* 0.001.

Results showed a significant direct effect of job insecurity on both WFC [*b*(se)=0.24 (0.05), *p*<0.001, *CI*s (0.11;0.30)] and burnout [*b*(se)=0.16 (0.03), *p*<0.001, *CI*s (0.04;0.13)], whereas no direct effect was found on job performance [*b*(se)=−0.08 (0.06), *p*=0.15, *CI*s (−0.21;0.03)]. At higher level of job insecurity, there is a significant positive association with higher level of WFC and burnout. Moreover, WFC showed a significant direct effect on burnout [*b*(se)=0.61 (0.03), *p*<0.001, *CI*s (0.31;0.40)] but no direct effect on job performance (*p*>0.05). Burnout, in turn, revealed a significant direct and negative effect on job performance [*b*(se)=−0.33, *p*<0.001, *CI*s (−1.34; −0.45)].

The analysis of the indirect effects (Table 4) indicated that burnout mediates the relationship between job insecurity and job performance [*b*(se)=0.05 (0.02), *CI*s (−0.10; −0.02]. In other words, job insecurity reported a positive association with job burnout that, in turn, is negatively related to employees’ job performance. Similarly, job insecurity is related to a greater perception of WFC, which is negatively associated with burnout and, in turn, job performance. Indeed, the serial mediation model was confirmed [*b*(se)=0.05 (0.02), *CI*s (−0.08; −0.02]. The direct effect analysis revealed a non-significant relationship between job insecurity and performance, thus suggesting a full-serial mediation model.

**Table 4:** Indirect, direct, and total effects of job insecurity on job performance.

	Effect	SE	95% Bootstrap CI
Indirect effect (INS→WFC→P)	−0.01	0.01	−0.04|0.03
Indirect effect (INS→B→P)	−0.05^*^	0.02	−0.10|−0.02
Indirect effect (INS→WFC→B→P)	−0.05^*^	0.02	−0.08|−0.02
Direct effect (INS→P)	−0.09	0.06	−0.21|0.03
Total effect (INS→P)	−0.11^***^	0.03	−0.16|−0.05

INS, job insecurity; WFC, Work-family conflict; B, burnout; P, job performance. CI, confidence interval and SE, standard error;

* *p<* 0.05; ^**^p< 0.01;

*** *p<* 0.001 or CI does not cross zero.

The predictors hypothesized in the serial mediation model covered approximately 16% of job performance variance (*R*^2^=0.16). Furthermore, the results confirm the mediating role of WFC on burnout, which, in turn, mediated the relationship between job insecurity and job performance. In other words, the greater the job insecurity, the greater the likelihood of experiencing a conflict between work and family demands. This discomfort becomes increasingly associated with burnout, which may ultimately deteriorate the employee’s job performance.

The hypothesized moderated serial mediation model was tested using Model 84 described by Hayes (2017), in which the independent variable (i.e., job insecurity) is directly associated with the dependent variable (i.e., job performance) and indirectly associated through a serial mediation relationship (i.e., WFC and burnout, respectively). In addition, the relationship between the independent variable and the two hypothesized mediators is moderated by an external variable (i.e., organizational justice). In this sense, the model explores job insecurity’s direct and indirect effects on job performance in an overall moderated serial mediation path. Organizational justice was hypothesized as a positive organizational factor that can buffer the negative effect of an uncertain labor market. Table 4 displays the standardized regression coefficients, standard errors (SE), and model summary information for the moderated serial mediation model of job insecurity on the perceived job performance.

Table 5 presents the results of the hypothesized moderated effect of organizational justice on the multiple mediated relationship between job insecurity and job performance. The regression coefficients showed a similar pattern to the previous model, with slight effect variations. In the additional hypothesized model, organizational justice showed a negative direct effect on both the mediators (i.e., WFC and job burnout). In other words, higher level of perceived organizational justice is significantly correlated with lower level of WFC [*B*(se)=−0.31 (0.06), *p*<0.001, *CI*s (−0.43; −0.19)] and lower level of burnout symptoms [*B*(se)=−0.14 (0.03), *p*<0.001, *CI*s (−0.20; −0.09)].

**Table 5:** Regression coefficients, SE, and model summary information for the moderated serial mediation model.

Antecedent	Consequent								
	WFC (M1)			Burnout (M2)			Job performance		
	Coeff.	SE	*p*	Coeff.	SE	*p*	Coeff.	SE	*p*
(Intercept)	2.88^***^	0.22	<0.001	1.14^***^	0.12	<0.001	9.52^***^	0.38	<0.001
Job insecurity	0.15^**^	0.05	< 0.01	0.07^**^	0.02	< 0.01	−0.09	0.06	0.15
WFC	–	–	–	0.32^***^	0.02	< 0.001	−0.03	0.08	0.70
Burnout	–	–	–				−0.74^***^	0.14	<0.001
Organizational justice	−0.31^***^	0.06	< 0.001	−0.14^***^	0.03	< 0.001			
INS × JUS	−0.11^*^	0.05	< 0.05	−0.04	0.02	0.09			
Age	−0.01	0.01	0.15	−0.01	0.01	0.90	0.01^*^	0.01	< 0.05
Gender (0=female)	−0.19	0.11	0.08	0.08	0.05	0.09	−0.21	0.14	0.14
Job tenure	0.01	0.01	0.90	0.01	0.01	0.60	−0.01	0.01	0.11
Job Contract (0=permanent)	−0.24	0.15	0.10	−0.03	0.07	0.63	0.19	0.19	0.31
	*R*^2^ =0.14 *F*_(7,341)_ =7.742 *p* <0.001			*R*^2^ =0.47 *F*_(8,341)_ =38.259 *p* <0.001			*R*^2^ =0.16 *F*_(9,340)_ =10.956 *p* <0.001		

INS × JUS, job insecurity × organizational justice. M1 (organizational justice on the relationship between job insecurity and WFC). M2 (organizational justice on the relationship between job insecurity and burnout);

* *p<* 0.05;

** *p<* 0.01;

*** *p<* 0.001.

**Figure 2:**
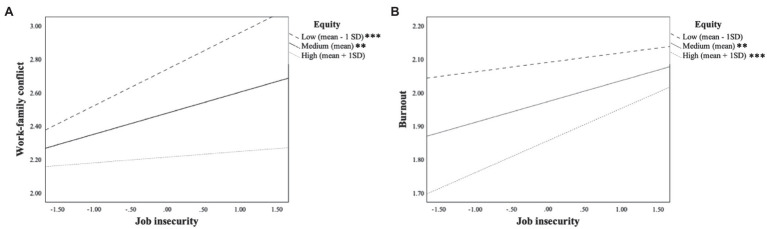
Conditional effects of low, medium, and high levels of equity on the relationship between **(A)** job insecurity and WFC and between **(B)** job insecurity and burnout.

The interaction between job insecurity and organizational justice was significant only on the first mediator (i.e., WFC) [*B*(se)=−0.11 (0.05), *p*<0.05; *CI*s (−0.22; −0.01)]. Nonetheless, the conditional direct and indirect effects analysis and indices for the moderated serial mediation model supported the buffering effect of organizational justice (Table 6). In more detail, the analysis of the conditional direct effects of the total predictors at values of the moderator (Table 7) revealed a significant effect at low [B(se) = 0.24 (0.07, p < 0.001; CIs (0.11; 0.37)] and medium level [B(se) = 0.15 (0.05), CIs (0.05; 0.25)] of organizational justice. The analysis further highlighted an interesting hampering effect at medium [*B*(se)=0.07 (0.02), *CI*s (0.02; 0.11)] and high level [*B*(se)=0.10 (0.03), *CI*s (0.04;0.16)] of organizational justice on the relationship between job insecurity and burnout. Figure 2 shows the conditional effects of low, medium, and high level of equity on the relationship between (1) job insecurity and WFC and between (2) job insecurity and burnout. Finally, the index of moderated mediation is a significant moderating effect of organizational justice on the serial mediation between job insecurity and job performance [*B*(se)=0.03 (0.02), *CI*s (0.01; 0.06)].

**Table 6:** Conditional direct, indirect effects, and indexes for the moderated serial mediation model.

Organizational Justice	Indirect effect (INS → WFC → JP)		Indirect effect (INS → B → JP)		Indirect effect (INS → WFC → B → JP)		Direct effect (INS → JP)		
	Effect	95% Bootstrap CI	Effect	95% Bootstrap CI	Effect	95% Bootstrap CI	Effect	SE	p
							−0.09	0.06	0.15
Low (−1 SD)	−0.01	−0.05|0.04	−0.02	−0.08|0.03	−0.02^*^	−0.11|−0.02			
Medium (mean)	−0.01	−0.03|0.02	−0.05^*^	−0.10|−0.01	−0.01^*^	−0.07|−0.01			
High (+1 SD)	−0.01	−0.02|0.01	−0.07^*^	−0.13|−0.03	−0.02	−0.05|0.01			
Index of moderated mediation	0.01	−0.01|0.03	−0.03	−0.08|0.01	0.03^*^	0.01|0.06			

INS, job insecurity; WFC, WFC; B, burnout; P, job performance; CI confidence interval and SE, standard errors;

* *p<* 0.05 or CI does not cross zero.

**Table 7:** The conditional direct effects of the focal predictor at values of the moderator.

Organizational justice	Direct effect (INS→WFC)			Direct effect (INS→B)		
	Effect	SE	95% Bootstrap CI	Effect	SE	95% Bootstrap CI
Low (−1 SD)	0.24^***^	0.06	0.11|0.37	0.03	0.03	−0.03|0.09
Medium (mean)	0.15^**^	0.05	0.06|0.25	0.07^**^	0.02	0.02|0.11
High (+1 SD)	0.06	0.07	−0.06|0.19	0.10^***^	0.03	0.04|0.16

INS, job insecurity; Work-family conflict, WFC; B, burnout; CI, confidence interval and SE, standard errors.

* *p<* 0.05; ^**^p< 0.01;

*** *p<* 0.001.

These results confirmed the moderated role of organizational justice on the serial association between WFC and burnout, which mediated the relationship between job insecurity and job performance. In other words, employees reporting a high level of job insecurity combined with a low level of perceived organizational justice in their work environment reported a more significant deterioration in their job performance.

## Discussion

The purpose of the current study was to extend knowledge of the relationship between job insecurity and job performance and provide a theoretical framework that accounts for the underlying variables involved in this relationship and its effects. Accordingly, the general aim of this study was to explore the serial mediating role of WFC and job burnout in the relationship between job insecurity and job performance. Additionally, a further goal was to test whether organizational justice could buffer the negative association of job insecurity with WFC and burnout.

During the last decades, characterized by the growing instability resulting from the rapid socioeconomic and technological changes in the labor market worldwide, job insecurity has gained significant relevance as one of the prominent antecedents of impaired job performance (Piccoli et al., 2021). Moreover, the subjective feeling of a threat to job stability seems progressively triggered by external contextual pressures on organizations, which are increasingly forced to react quickly by changing processes, structures, and activities (Lee et al., 2018). The recent pandemic has severely strained various occupational sectors, many companies have experienced downsizing, many more have closed, and unemployment has risen dramatically (Rudolph et al., 2020). These dynamics raise crucial questions about the impact of such insecurity on job performance, given past ambiguous findings on the topic that have not helped untangle this issue (Debus et al., 2019).

The current study provided additional insights on the relationship between job insecurity and job performance, highlighting the potential mechanisms explaining how performance might be impaired due to inadequate individual and organizational responses. The obtained results suggested that job insecurity was negatively related to job performance, thus corroborating the assumption that employees repetitively exposed to a perceived threat on their job’s stability (e.g., threats outside the organization) cannot carry out their work tasks successfully. Also, the indirect nature of such a relationship might be an exciting approach to adopt in future research to deepen the understanding of how job insecurity affects job performance, thus potentially overcoming the inconsistent findings gathered so far (Lee et al., 2018). How organizations respond, adapt, and react to external environment disruptions can impact the organization (e.g., procedures, sustainability, and economics) and the individual. The results of this study are even more interesting if viewed in the aftermath of the pandemic outbreak. Many have recognized the pandemic as a significant stress factor for organizations and individuals (Rigotti et al., 2021). Forced remote work may have led to experiencing the transition negatively, with feelings of technostress (Molino et al., 2020), little support from the supervisors (Vaziri et al., 2020), which in turn, may have negatively affected how people manage their family responsibilities, as our results might suggest. Based on our results, it is not unreasonable to hypothesize that levels of job insecurity may have reached unsustainable peaks during the pandemic that could have had a considerable impact on the work-family balance, ultimately deteriorating job performance. Milliken et al. (2020) also discussed that the shift to remote work during the pandemic might have dissolved the boundaries between work and family, creating new gender dynamics. Despite the greater presence of women in the sample, the hypothesized model involved both genders, suggesting how the job insecurity, already present in the aftermath of the pandemic, may have exacerbated WFC in the months that followed where both partners might have shared the working remotely together. Different needs for childcare, housework, and longer working days might suggest the need for organizational support in this direction.

Indeed, in the occupational health field, job insecurity has also been a concern of most companies and academic research due to its critical impact on employee wellbeing (Richter et al., 2014), burnout, and emotional exhaustion (De Cuyper et al., 2012; Jiang and Probst, 2017). Building on this, the present findings revealed that job burnout mediated the association between job insecurity and performance. In other words, employees perceiving the future of their current job position as highly uncertain also experience a more significant occurrence of burnout symptoms and, consequently, a reduced ability to perform properly at work. The mediating role of burnout agrees with earlier empirical findings that identified job insecurity as one of the most substantial hindrance stressors that deplete employees’ energies and resources, thus progressively undermining employees’ wellbeing (Darvishmotevali and Ali, 2020). Accordingly, the negative relationship between job insecurity and performance was mediated by burnout symptoms in the present study. As suggested by Piccoli et al. (2021), uncertainty derived from the fear of losing the job seems more likely to be experienced as a hindering stressor. From the results of the present study, a mechanism could thus be delineated whereby the emergence of a feeling of job insecurity experienced as an impeding factor is predominantly associated with negative emotions and attitudes (e.g., burnout) that may ultimately lead to behavioral withdrawal and passive coping strategies (e.g., impaired performance) rather than reactive coping strategies.

Our findings suggest that employees perceiving solid levels of job insecurity are more prone to experience a higher WFC, which is related to a greater occurrence of burnout symptoms and, subsequently, decreased job performance. The full-serial mediation opens interesting future research by considering the way job insecurity is experienced at home as a key mechanism influencing people’s health and wellbeing, and thus indirectly their work performance. People may experience this uncertainty even more strongly given the potential negative impact on work-family balance, thus undermining the health of those involved. A vicious circle in which energy is drained, and performance is eventually reduced. In other words, this contribution could provide further insights into the underlying conceptual dimensions of work performance, considering factors located at different levels of analysis (i.e., environmental, organizational, and individual).

In detail, external environmental pressures define the potential degree of organizational instability and perceived job insecurity. It should be noted that in this sample, more than half declared to be employed with a permanent contract. Nevertheless, the hypothesized model is significant, suggesting a potentially pivotal role of the environment. Concerning this point, the organization might play a crucial role on two fronts. On the one hand, the way the organization reacts to external challenges and pressures defines its success and, conversely, the degree of insecurity perceived by its employees (i.e., job insecurity).

On the other hand, the way the organization internally manages, explains, and clarifies to employees its response to the external challenges plays a crucial role in terms of perceived organizational justice. For example, Rodwell and Gulyas (2015) highlighted how the clarity and transparency of the process behind breaches of the psychological contract allowed employees to understand the reasons for such breaches, accepting the consequences. In other words, the level of transparency or perceived fairness in the information shared and explanation given about organizational decisions are processes that organizations cannot ignore.

Especially in the face of the “new normal” emphasized by the COVID-19 pandemic, this study may suggest that even if forced to make difficult choices due to external pressures or constraints that could undermine employees’ job security, the organization is perceived as fair if it puts in place clear and transparent processes to explain these choices. This can protect employees’ work-family relationships and mental health and ultimately prevent reduced job performance. How the individual perceives and interprets these external pressures and the organization’s strategies play a crucial role in the overall hypothesized process. The greater the perceived job insecurity, the greater the likelihood that employees will experience this situation negatively, thus creating a vicious and potentially draining cycle over time. The role of an organization perceived as fair and transparent can only mitigate the uncertainties of an unstable and sometimes unpredictable environment, as was the case during the pandemic.

In conclusion, these results show that perceived job insecurity could lead to a lower ability to manage the family role. In line with previous findings (Mauno et al., 2017), the spillover effect transfers the fear of job loss within the family context, thus promoting work-family imbalance (Nauman et al., 2020). The present study suggests further insights to the literature by highlighting how the relationship between job insecurity and performance can be influenced by a detrimental mechanism that initially propagates at the individual level, affecting the work-family balance, and later impacts the mental health of individuals. In this case, the significant serial mediation model suggests that the risk of job-related outcomes (e.g., reduced performance) cannot be excluded.

Further evidence of the current investigation involves the buffering role of organizational justice as a protective organizational factor that can weaken the WFC and job burnout experienced by those employees perceiving a significant level of job insecurity. Findings suggest that perceived high levels of organizational justice may reduce the strength of the association between job insecurity and WFC. The role that an organization and its managers can play in a climate of uncertainty becomes hugely influential in terms of its impact on employees’ private lives (Kyei-Poku, 2014; Nauman et al., 2020; Sánchez et al., 2020). Indeed, in this study, the sense of being part of a fair working environment appears to play a crucial protective role in employees’ ability to balance their efforts between private and work life. In this sense, in a climate of uncertainty about own working and economic future, the possibility of relying on the management, capable of initiating positive social exchanges or available to share the criteria used to evaluate the performance of employees, seems to promote employee’s ability to positively deal with such a scenario, both at work and home.

On the other hand, the current results indicate that the detrimental association between job insecurity and burnout symptoms is solid when employees perceive their organizational setting as unfair, thus characterized by an inadequate level of justice. This result contributes to the current understanding of organizational justice as a workplace characteristic related to significant outcomes regarding employees’ attitudes and behaviors in the work environment (e.g., Yang et al., 2014). Consistent with previous studies, the more employees perceive an organizational environment that provides fair treatment, the less the condition of job insecurity translates into adverse individual and professional outcomes (Chirumbolo et al., 2020).

### Study Limitations and Directions for Further Research

The current study has some limitations that should be acknowledged. The main weakness comes from employing a cross-sectional design to assess a serial mediation model, thus preventing the opportunity to draw definite conclusions on the causal link explaining the relationships among the study variables. Hence, future studies could replicate the hypothesized model using a longitudinal research design with different measurement time points. These data could provide robust evidence of the causal impact of perceived job insecurity on WFC, burnout symptoms, and the resulting impairment of employees’ job performance, thus deepening the understanding of both the health-impairment process and the spillover effect.

An additional limitation of the current research lies on our data, which are based on self-reported measures. This choice could have enhanced the likelihood of common method variance effects among the variables under investigation (Podsakoff et al., 2003). Nevertheless, this limitation should be considered with prudence. First, recent research suggests that Common Method Bias (CMB) due to self-reports is not necessarily a problem and is sometimes overestimated (Brannick et al., 2010). In particular, WFC refers to the perceived difficulties in managing multiple demands stemming from one’s roles in the work domain and private life simultaneously, thus experiencing a perceived imbalance between these life spheres (Fotiadis et al., 2019).

Similarly, job burnout represents a psychological condition characterized by severe and persistent exhaustion and related symptoms, mainly referred to as mental distancing from work and cognitive-emotional impairment. Therefore, subjective measures could be considered the most reasonable and proper way to evaluate these constructs. Furthermore, it has been noted that several methods used to tackle CMB do not have the desired effects, and none answer the question definitively (Spector et al., 2019). Nevertheless, Harman’s single factor showed that one factor explained less than 50% of the variance (i.e., 27.4%), suggesting a non-significance of the CMB (Podsakoff et al., 2003). In contrast, future research that would replicate the current study design adopting objective measures of job insecurity and job performance are highly encouraged.

As a further limitation, in the present study, job insecurity was assessed exclusively in terms of quantitative job insecurity, which entails the perceived threat of losing one’s job (Låstad et al., 2015). In order to extend the current findings, future research implies testing the model also including a measure of qualitative job insecurity is also highly encouraged. Qualitative job insecurity is defined as the employees’ concerns over loss of valued conditions of the employment relationship, such as career development opportunities and the allocation of stimulating work tasks (Chirumbolo et al., 2020). This line of research would assess whether different facets of job insecurity exhibit a diverse impact on employees’ wellbeing and work outcomes.

Moreover, the current study assessed organizational justice as an overall concept through a unidimensional scale. On the other hand, academic literature recognizes several dimensions of organizational justice: procedural, distributive, and interpersonal justice (Cropanzano et al., 2007). Future studies could delve deeper into the current results by exploring whether organizational justice’s protective role could vary across different construct’ components. This focus would narrow the design of organizational interventions by targeting those aspects of justice more likely to deter harmful outcomes (i.e., WFC and burnout symptoms) among employees.

### Practical Implications

The obtained findings contribute to the current understanding of organizational justice’s protective role and might help developing related interventions. Our results suggest that organizational justice can prevent employees’ inadequate responses to job insecurity from deteriorating work-family relationships and mental health.

Accordingly, employees working in an organization that is embedded in an unpredictable environment but promotes organizational fairness are less likely to experience related harmful outcomes and more likely to adequately perform the assigned tasks (i.e., job performance). These findings suggest that organizational justice must be carefully considered in the implementation of HRM practices. For instance, companies should provide clear guidelines on the organizational goals that employees are supposed to meet and the procedures to evaluate their performance. A further strategy entails allocating suitable rewards and compensation for employees’ performance as an outcome of fair and equal procedures that guarantees equal access to these rewards (Bryant and Allen, 2013). The present findings become even more interesting given the pandemic’s impact on individuals and organizations. In a climate of uncertainty in which even organizations and managers may not have an adequate and ready counterstrategy to deal with the unexpected and continuous changes induced by the external context, adopting an approach based on transparency, fairness, and clarity in communication, allocation of resources and interpersonal relations could prove successful in safeguarding the health and performance of their employees. Through role modeling, managers and supervisors might play a pivotal role in protecting the employees’ work-family balance and performance. A family-friendly culture might mitigate stressors (i.e., job insecurity) and strains (Meyer et al., 2021) and increase perceived support from the organization and supervisor (French and Shockley, 2020). As Vaziri et al. (2020) suggested, organizations and managers who support and provide tips to handle the boundaries between work and home to employees (i.e., interactional justice) might prevent a lower level of job satisfaction and job performance and higher turnover intent.

Moreover, as also suggested by Giorgi et al. (2020), providing clear and rapid information (i.e., informational justice) on how to deal with the pandemic scenario and its consequences (even if hostile) about their daily job activities and their mental health may prove more effective than avoiding sharing such information. Also, providing organizational resources, such as adequate protection or equipment for working from home to all employees (i.e., distributive justice), could prove to be a compelling investment in preserving employees’ mental health and performance. Stanhope and Weinstein (2021) introduced that working with employees to find the most suitable solutions and procedures to manage workload or prioritize all available resources (i.e., procedural justice) should be considered a winning strategy instead of a barrier.

These measures are crucial among workers experiencing a great fear of job loss. Organizations and management that are receptive to employees’ concerns (e.g., job insecurity) become an essential strategy for maintaining a high level of employee engagement and commitment to work (Wang et al., 2015). Therefore, organizations providing a significant involvement in decision-making processes can help workers manage their uncertainty, enabling them to remain competitive and perform accordingly, particularly in complex and uncertain times as experienced nowadays. The ways organizations treat their employees during these times will have a crucial impact on their future (Rudolph et al., 2020).

## Data Availability Statement

The raw data supporting the conclusions of this article will be made available under request by the first author.

## Ethics Statement

Ethical review and approval were not required for the study on human participants in accordance with the local legislation and institutional requirements. The patients/participants provided their written informed consent to participate in this study.

## Author Contributions

MDA and GM contributed to the conceptualization, methodology, and writing of the original draft. MDA contributed to the formal analysis. MDA, GM, and DG contributed to the investigation and writing. DG contributed to the review and editing. All authors contributed to the manuscript and approved the submitted version.

## Funding

This paper has received funding from the European Union’s Horizon 2020 research and innovation programme under the project H-WORK—Multilevel Interventions to Promote Mental Health in SMEs and Public Workplaces (grant agreement no. 847386). The material presented and views expressed here are the responsibility of the authors only.

## Conflict of Interest

The authors declare that the research was conducted in the absence of any commercial or financial relationships that could be construed as a potential conflict of interest.

## Publisher’s Note

All claims expressed in this article are solely those of the authors and do not necessarily represent those of their affiliated organizations, or those of the publisher, the editors and the reviewers. Any product that may be evaluated in this article, or claim that may be made by its manufacturer, is not guaranteed or endorsed by the publisher.

## References

[ref1] Aiken L. S. West S. G. (1991). Multiple Regression: Testing and Interpreting Interactions. Newbury Park: Sage.

[ref2] Avallone F. Paplomatas A. (2005). Psicologia del benessere nei contesti lavorativi. Milano: Raffaello Cortina Editore.

[ref3] Bakker A. B. Demerouti E. (2014). “Job demands-resources theory,” in Work and Wellbeing: Wellbeing: A Complete Reference Guide. *Vol*. 3. eds. Chen P. Y. Cooper C. L. (New York: John Wiley and Sons), 1–28.

[ref4] Bakker A. B. Demerouti E. (2017). Job demands-resources theory: taking stock and looking forward. J. Occup. Health Psychol. 22, 273–285. 10.1037/ocp0000056, PMID: 27732008

[ref5] Benach J. Vives A. Amable M. Vanroelen C. Tarafa G. Muntaner C. (2014). Precarious employment: understanding an emerging social determinant of health. Annu. Rev. Public Health 35, 229–253. 10.1146/annurev-publhealth-032013-182500, PMID: 24641559

[ref6] Berntson E. Näswall K. Sverke M. (2010). The moderating role of employability in the association between job insecurity and exit, voice, loyalty and neglect. Econ. Ind. Democracy 31, 215–230. 10.1177/0143831X09358374

[ref7] Blustein D. L. Duffy R. Ferreira J. A. Cohen-Scali V. Cinamon R. G. Allan B. A. (2020). Unemployment in the time of COVID-19: A research agenda. J. Vocat. Behav. 119:103436. 10.1016/j.jvb.2020.103436, PMID: 32390656 PMC7206417

[ref8] Brannick M. T. Chan D. Conway J. M. Lance C. E. Spector P. E. (2010). What is method variance, and how can we cope with it? A panel discussion. Organ. Res. Methods 13, 407–420. 10.1177/1094428109360993

[ref9] Bryant P. C. Allen D. G. (2013). Compensation, benefits and employee turnover: HR strategies for retaining top talent. Compens. Benefits Rev. 45, 171–175. 10.1177/0886368713494342

[ref10] Byron K. (2005). A meta-analytic review of work-family conflict and its antecedents. J. Vocat. Behav. 67, 169–198. 10.1016/j.jvb.2004.08.009

[ref11] Cheng G. H. L. Chan D. K. S. (2008). Who suffers more from job insecurity? A meta-analytic review. Appl. Psychol. 57, 272–303. 10.1111/j.1464-0597.2007.00312.x

[ref12] Chirumbolo A. Callea A. Urbini F. (2020). Job insecurity and performance in public and private sectors: a moderated mediation model. J. Organ. Eff. People Perform. 7, 237–253. 10.1108/JOEPP-02-2020-0021

[ref13] Chirumbolo A. Hellgren J. (2003). Individual and organizational consequences of job insecurity: A European study. Econ. Ind. Democracy 24, 217–240. 10.1177/0143831X03024002004

[ref14] Cohen J. (1988). Statistical Power Analysis for the Behavioral Sciences. 2nd *Edn*. Hillsdale, NJ: Laurence and Erlbaum.

[ref15] Cropanzano R. Bowen D. E. Gilliland S. W. (2007). The management of organizational justice. Acad. Manag. Perspect. 21, 34–48. 10.5465/amp.2007.27895338

[ref16] Darvishmotevali M. Ali F. (2020). Job insecurity, subjective well-being and job performance: The moderating role of psychological capital. Int. J. Hosp. Manag. 87:102462. 10.1016/j.ijhm.2020.102462

[ref17] De Cuyper N. De Witte H. (2005). Job insecurity: mediator or moderator of the relationship between type of contract and various outcomes. SA J. Ind. Psychol. 31, 79–86. 10.4102/sajip.v31i4.211

[ref18] De Cuyper N. Mäkikangas A. Kinnunen U. Mauno S. Witte H. D. (2012). Cross-lagged associations between perceived external employability, job insecurity, and exhaustion: testing gain and loss spirals according to the conservation of resources theory. J. Organ. Behav. 33, 770–788. 10.1002/job.1800

[ref19] De Witte H. (2005). Job insecurity: review of the international literature on definitions, prevalence, antecedents and consequences. J. Ind. Psychol. 31, 1–6. 10.4102/sajip.v31i4.200

[ref20] De Witte H. Pienaar J. De Cuyper N. (2016). Review of 30 years of longitudinal studies on the association between job insecurity and health and well-being: is there causal evidence? Aust. Psychol. 51, 18–31. 10.1111/ap.12176

[ref21] De Witte H. Vander Elst T. De Cuyper N. (2015). “Job insecurity, health and well-being,” in Sustainable Working Lives. eds. Vuori J. Blonk R. H. Price (Dordrecht, NY: Springer Science + Business Media), 109–128.

[ref22] Debus M. E. Unger D. König C. J. (2019). Job insecurity and performance over time: the critical role of job insecurity duration. Career Dev. Int. 25, 325–336. 10.1108/CDI-04-2018-0102

[ref23] Di Stefano G. Venza G. Aiello D. (2020). Associations of job insecurity With perceived work-related symptoms, job satisfaction, and turnover intentions: The mediating role of leader–member exchange and the moderating role of organizational support. Front. Psychol. 11:1329. 10.3389/fpsyg.2020.01329, PMID: 32733309 PMC7358553

[ref24] Fisher J. Languilaire J. C. Lawthom R. Nieuwenhuis R. Petts R. J. Runswick-Cole K.. (2020). Community, work, and family in times of COVID-19. Community Work Fam. 23, 247–252. 10.1080/13668803.2020.1756568

[ref25] Fotiadis A. Abdulrahman K. Spyridou A. (2019). The mediating roles of psychological autonomy, competence and relatedness on work-life balance and well-being. Front. Psychol. 10:1267. 10.3389/fpsyg.2019.01267, PMID: 31191420 PMC6549400

[ref26] French K. A. Shockley K. M. (2020). Formal and informal supports for managing work and family. Curr. Dir. Psychol. Sci. 29, 207–216. 10.1177/0963721420906218

[ref27] Gerstel N. Clawson D. (2018). Control over time: employers, workers, and families shaping work schedules. Annu. Rev. Sociol. 44, 77–97. 10.1146/annurev-soc-073117-041400

[ref28] Gilboa S. Shirom A. Fried Y. Cooper C. (2008). A meta-analysis of work demand stressors and job performance: examining main and moderating effects. Pers. Psychol. 61, 227–271. 10.1111/j.1744-6570.2008.00113.x

[ref29] Giorgi G. Lecca L. I. Alessio F. Finstad G. L. Bondanini G. Lulli L. G.. (2020). COVID-19-related mental health effects in the workplace: A narrative review. Int. J. Environ. Res. Public Health 17:7857. 10.3390/ijerph17217857, PMID: 33120930 PMC7663773

[ref30] Giunchi M. Vonthron A. M. Ghislieri C. (2019). Perceived job insecurity and sustainable wellbeing: do coping strategies help? Sustain 11:784. 10.3390/su11030784

[ref31] Grandey A. A. Cordeiro B. L. Michael J. H. (2007). Work-family supportiveness organizational perceptions: important for the well-being of male blue-collar hourly workers? J. Vocat. Behav. 71, 460–478. 10.1016/j.jvb.2007.08.001

[ref32] Green F. (2011). Unpacking the misery multiplier: how employability modifies the impacts of unemployment and job insecurity on life satisfaction and mental health. J. Health Econ. 30, 265–276. 10.1016/j.jhealeco.2010.12.005 21236508

[ref33] Griep Y. Lukic A. Kraak J. M. Bohle S. A. L. Jiang L. Vander Elst T.. (2021). The chicken or the egg: The reciprocal relationship between job insecurity and mental health complaints. J. Bus. Res. 126, 170–186. 10.1016/j.jbusres.2020.12.045

[ref34] Guglielmi D. Depolo M. Simbula S. Paplomatas A. (2011). Prevenzione dello stress lavoro correlato: validazione di uno strumento per la valutazione dei rischi psicosociali nella scuola. Prevenzione dello stress lavoro correlato: validazione di uno strumento per la valutazione dei rischi psicosociali nella scuola. Psicol. Salute 3, 53–74. 10.3280/PDS2011-003003

[ref35] Hayes A. F. (2015). An index and test of linear moderated mediation. Multivar. Behav. Res. 50, 1–22. 10.1080/00273171.2014.962683, PMID: 26609740

[ref36] Hayes A. F. (2017). Introduction to Mediation, Moderation, and Conditional Process Analysis: A Regression-Based Approach. New York, NY: Guilford publications.

[ref37] Hu S. Jiang L. Probst T. M. Liu M. (2018). The relationship between qualitative job insecurity and subjective well-being in Chinese employees: The role of work-family conflict and work centrality. Econ. Ind. Democracy 40, 203–225. 10.1177/0143831X18759793

[ref38] Jiang L. Lavaysse L. M. (2018). Cognitive and affective job insecurity: A meta-analysis and a primary study. J. Manage. 44, 2307–2342. 10.1177/2F0149206318773853

[ref39] Jiang L. Probst T. M. (2017). The rich get richer and the poor get poorer: country-and state-level income inequality moderates the job insecurity-burnout relationship. J. Appl. Psychol. 102, 672–681. 10.1037/apl0000179, PMID: 27893258

[ref40] Kyei-Poku I. A. (2014). Linking interactional justice to work-to-family conflict: the mediating role of emotional exhaustion. Organ. Manag. J. 11, 74–83. 10.1080/15416518.2014.929932

[ref41] Låstad L. Berntson E. Näswall K. Lindfors P. Sverke M. (2015). Measuring quantitative and qualitative aspects of the job insecurity climate. Career Dev. Int. 20, 202–217. 10.1108/CDI-03-2014-0047

[ref42] László K. D. Pikhart H. Kopp M. S. Bobak M. Pajak A. Malyutina S.. (2010). Job insecurity and health: A study of 16 European countries. Soc. Sci. Med. 70, 867–874. 10.1016/j.socscimed.2009.11.022, PMID: 20060634 PMC2845821

[ref43] Lazarus R. S. Folkman S. (1984). “Coping and adaptation,” in The Handbook of Behavioral Medicine. ed. Gentry W. D. (New York: Guilford), 282–325.

[ref44] Lee C. Huang G. H. Ashford S. J. (2018). Job insecurity and the changing workplace: recent developments and future trends in job insecurity research. Ann. Rev. Organ. Psychol. Organ. Behav. 5, 335–359. 10.1146/annurev-orgpsych-032117-104651

[ref45] Mauno S. Cheng T. Lim V. (2017). The far-reaching consequences of job insecurity: A review on family-related outcomes. Marriage Fam. Rev. 53, 717–743. 10.1080/01494929.2017.1283382

[ref46] Mauno S. Kinnunen U. Ruokolainen M. (2007). Job demands and resources as antecedents of work engagement: A longitudinal study. J. Vocat. Behav. 70, 149–171. 10.1016/j.jvb.2006.09.002

[ref47] McKibbin W. J. Fernando R. (2020). *The Global Macroeconomic Impacts of COVID-19: Seven Scenarios*: March 20, 2020. CAMA Working Paper No. 19/202.

[ref48] Menéndez-Espina S. Llosa J. A. Agulló-Tomás E. Rodríguez-Suárez J. Sáiz-Villar R. Lahseras-Díez H. F. (2019). Job insecurity and mental health: The moderating role of coping strategies from a gender perspective. Front. Psychol. 10:286. 10.3389/fpsyg.2019.00286, PMID: 30833919 PMC6387966

[ref49] Menéndez-Espina S. Llosa J. A. Agulló-Tomás E. Rodríguez-Suárez J. Sáiz-Villar R. Lasheras-Díez H. F.. (2020). The influence of gender inequality in the development of job insecurity: differences between women and men. Front. Public Health 8:526162. 10.3389/fpubh.2020.526162, PMID: 33163470 PMC7581853

[ref50] Meyer B. Zill A. Dilba D. Gerlach R. Schumann S. (2021). Employee psychological well-being during the COVID-19 pandemic in Germany: A longitudinal study of demands, resources, and exhaustion. Int. J. Psychol. 53, 532–550. 10.1002/ijop.12743, PMID: 33615477 PMC8013458

[ref51] Milliken F. J. Kneeland M. K. Flynn E. (2020). Implications of the COVID-19 pandemic for gender equity issues at work. J. Manag. Stud. 57, 1767–1772. 10.1111/joms.12628

[ref52] Molino M. Ingusci E. Signore F. Manuti A. Giancaspro M. L. Russo V.. (2020). Wellbeing costs of technology use during covid-19 remote working: an investigation using the Italian translation of the technostress creators scale. Sustainability 12:5911. 10.3390/su12155911

[ref53] Mutambudzi M. Javed Z. Kaul S. Prochaska J. Peek M. K. (2017). Effects of work-family conflict and job insecurity on psychological distress. Occup. Med. 67, 637–640. 10.1093/occmed/kqx067 28535248

[ref54] Nauman S. Zheng C. Naseer S. (2020). Job insecurity and work-family conflict: A moderated mediation model of perceived organizational justice, emotional exhaustion and work withdrawal. Int. J. Confl. Manag. 31, 729–751. 10.1108/IJCMA-09-2019-0159

[ref55] Parent-Lamarche A. Marchand A. Saade S. (2021). How do work organization conditions affect job performance? The mediating role of workers’ well-being. J. Workplace Behav. Health. 38, 48–76. 10.1080/15555240.2021.1872382

[ref56] Piccoli B. De Witte H. (2015). Job insecurity and emotional exhaustion: testing psychological contract breach versus distributive injustice as indicators of lack of reciprocity. Work Stress. 29, 246–263. 10.1080/02678373.2015.1075624

[ref57] Piccoli B. Reisel W. D. De Witte H. (2021). Understanding the relationship between job insecurity and performance: hindrance or challenge effect? J. Career Dev. 48, 150–165. 10.1177/0894845319833189

[ref58] Pilipiec P. (2020). The role of time in the relation between perceived job insecurity and perceived job performance. Work 66, 3–15. 10.3233/WOR-203145, PMID: 32417808 PMC7369069

[ref59] Podsakoff P. M. MacKenzie S. B. Lee J. Y. Podsakoff N. P. (2003). Common method biases in behavioral research: a critical review of the literature and recommended remedies. J. Appl. Psychol. 88, 879–903. 10.1037/0021-9010.88.5.879, PMID: 14516251

[ref60] Rasdi R. M. Zaremohzzabieh Z. Ahrari S. (2021). Financial insecurity During the COVID-19 pandemic: Spillover effects on burnout–disengagement relationships and performance of employees who moonlight. Front. Psychol. 12:610138. 10.3389/fpsyg.2021.610138, PMID: 33679526 PMC7929995

[ref61] Richter A. Näswall K. Bernhard-Oettel C. Sverke M. (2014). Job insecurity and well-being: The moderating role of job dependence. Eur. J. Work Organ. Psychol. 23, 816–829. 10.1080/1359432X.2013.805881

[ref62] Richter A. Näswall K. Sverke M. (2010). Job insecurity and its relation to work-family conflict: mediation with a longitudinal data set. Econ. Ind. Democracy 31, 265–280. 10.1177/0143831X09358370

[ref63] Rigotti T. Yang L. Q. Jiang Z. Newman A. De Cuyper N. Sekiguchi T. (2021). Work-related psychosocial risk factors and coping resources during the COVID-19 crisis. Appl. Psychol. 70, 3–15. 10.1111/apps.12307, PMID: 33821077 PMC8014633

[ref64] Rocha C. Hause Crowell J. McCarter A. K. (2006). The effects of prolonged job insecurity on the psychological well-being of workers. J. Sociol. Soc. Welfare 33, 9–28.

[ref65] Rodwell J. Gulyas A. (2015). Psychological contract breach among allied health professionals: fairness, individual differences and an aggravated breach effect. J. Health Organ. Manag. 29, 393–412. 10.1108/JHOM-05-2013-0107, PMID: 25970532

[ref66] Rudolph C. W. Allan B. Clark M. Hertel G. Hirschi A. Kunze F.. (2020). Pandemics: implications for research and practice in industrial and organizational psychology. Ind. Organ. Psychol. Perspect. Sci. Pract. 14, 1–35. 10.31234/osf.io/k8us2

[ref67] Sánchez I. D. Andrade J. M. Losada-Otálora M. (2020). Beyond organisational boundaries: the complex relationship between transformational leadership, organisational justice, and work-family conflict. Int. J. Hum. Resour. Dev. Manag. 20, 322–348. 10.1504/IJHRDM.2020.107990

[ref68] Schaufeli W. B. Desart S. De Witte H. (2020). Burnout assessment tool (BAT)-development, validity, and reliability. Int. J. Environ. Res. Public Health 17:9495. 10.3390/ijerph17249495, PMID: 33352940 PMC7766078

[ref69] Schaufeli W. B. Taris T. W. (2014). A critical review of the job demands–resources model: implications for improving work and health,” in Bridging Occupational, Organizational and Public health. eds. Bauer G. F. Hämmig O. (Dordrecht, The Netherlands: Springer), 43–68.

[ref70] Schumacher D. Schreurs B. De Cuyper N. Grosemans I. (2020). The ups and downs of felt job insecurity and job performance: The moderating role of informational justice. Work Stress. 35, 1–22. 10.1080/02678373.2020.1832607

[ref71] Shimazu A. Schaufeli W. B. Taris T. (2010). How does workaholism affect worker health and performance? The mediating role of coping. Int. J. Behav. Med. 17, 154–160. 10.1007/s12529-010-9077-x, PMID: 20169433

[ref72] Shin Y. Hur W. M. (2020). When do job-insecure employees keep performing well? The buffering roles of help and prosocial motivation in the relationship between job insecurity, work engagement, and job performance. J. Bus. Psychol. 36, 659–678. 10.1007/s10869-020-09694-4

[ref73] Shoss M. K. (2017). Job insecurity: An integrative review and agenda for future research. J. Manag. 43, 1911–1939. 10.1177/0149206317691574

[ref100] Silla I. Gracia F. J. Mañas M. A. Peiró J. M. (2010). Job insecurity and employees’ attitudes: the moderating role of fairness. Int. J. Manpower 31, 449–465. 10.1108/01437721011057029

[ref74] Sora B. Caballer A. Peiró J. M. Silla I. Gracia F. J. (2010). Moderating influence of organizational justice on the relationship between job insecurity and its outcomes: A multilevel analysis. Econ. Ind. Democracy 31, 613–637. 10.1177/0143831X10365924

[ref75] Sora B. Höge T. Caballer A. Peiró J. M. Boada J. (2021). Job insecurity and performance: The mediating role of organizational justice in terms of type of contract. [Inseguridad y desempeño laboral: El papel mediador de la justicia organizacional considerando el tipo de contrato]. Psicothema 33, 86–94. 10.7334/psicothema2020.205 33453740

[ref76] Spector P. E. Rosen C. C. Richardson H. A. Williams L. J. Johnson R. E. (2019). A new perspective on method variance: A measure-centric approach. J. Manag. 45, 855–880. 10.1177/2F0149206316687295

[ref77] Stanhope J. Weinstein P. (2021). Organisational injustice from the COVID-19 pandemic: a hidden burden of disease. Perspect. Public Health 141, 13–14. 10.1177/1757913920959113 33369544

[ref78] Stankevičiūtė Ž. Staniškienė E. Ramanauskaitė J. (2021). The impact of job insecurity on organisational citizenship behaviour and task performance: evidence from robotised furniture sector companies. Int. J. Environ. Res. Public Health 18, 1–17. 10.3390/ijerph18020515 PMC7827618 33435183

[ref79] Stynen D. Forrier A. Sels L. De Witte H. (2015). The relationship between qualitative job insecurity and OCB: differences across age groups. Econ. Ind. Democracy 36, 383–405. 10.1177/0143831X13510326

[ref80] Sverke M. Hellgren J. Näswall K. (2002). No security: a meta-analysis and review of job insecurity and its consequences. J. Occup. Health Psychol. 7, 242–264. 10.1037/1076-8998.7.3.242, PMID: 12148956

[ref81] Sverke M. Hellgren J. Näswall K. (2006). Job Insecurity: A Literature Review. Joint Programme for Working Life Research in Europe (SALTSA). Stockholm, SW: National Institute for Working Life. 1–30.

[ref83] Van den Broeck A. Sulea C. Vander Elst T. Fischmann G. Iliescu D. De Witte H. (2014). The mediating role of psychological needs in the relation between qualitative job insecurity and counterproductive work behavior. Career Dev. Int. 19, 526–547. 10.1108/CDI-05-2013-0063

[ref84] Vaziri H. Casper W. J. Wayne J. H. Matthews R. A. (2020). Changes to the work-family interface during the COVID-19 pandemic: examining predictors and implications using latent transition analysis. J. Appl. Psychol. 105, 1073–1087. 10.1037/apl0000819, PMID: 32866024

[ref85] Wang H. J. Lu C. Q. Siu O. L. (2015). Job insecurity and job performance: The moderating role of organizational justice and the mediating role of work engagement. J. Appl. Psychol. 100, 1249–1258. 10.1037/a0038330, PMID: 25402953

[ref86] Wilson J. M. Lee J. Fitzgerald H. N. Oosterhoff B. Sevi B. Shook N. J. (2020). Job insecurity and financial concern during the COVID-19 pandemic are associated with worse mental health. *J*. *Occup. Environ. Med*. 62, 686–691. 10.1097/JOM.0000000000001962, PMID: 32890205

[ref87] World Medical Association (2013). Declaration of Helsinki: ethical principles for medical research involving human subjects. J. Am. Med. Assoc. 310, 2191–2194. 10.1001/jama.2013.281053 24141714

[ref88] Yang L. Q. Bauer J. Johnson R. E. Groer M. W. Salomon K. (2014). Physiological mechanisms that underlie the effects of interactional unfairness on deviant behavior: The role of cortisol activity. J. Appl. Psychol. 99, 310–332. 10.1037/a0034413, PMID: 24099347

[ref89] Yeves J. Bargsted M. Cortes L. Merino C. Cavada G. (2019). Age and perceived employability as moderators of job insecurity and job satisfaction: A moderated moderation model. Front. Psychol. 10:799. 10.3389/fpsyg.2019.00799, PMID: 31031675 PMC6473047

